# Valedictory Address to the Graduating Class of Rush Medical College, Session 1862-3

**Published:** 1863-02

**Authors:** R. L. Rea


CHICAGO
MEDICAL JOURNAL.

VOL. VI.] FEBRUARY, 1863. [No. 2.

### ORIGINAL COMMUNICATIONS.

### VALEDICTORY ADDRESS

### TO THE GRADUATING CLASS OF RUSH MEDICAL COLLEGE, SESSION 1862-3.

By Prof. R. L. REA, M. D.

GENTLEMEN GRADUATES: — By the generous courtesy of my colleagues, I have the honor, as well as the very great pleasure of offering you our congratulations and saying the parting words.

It is most natural that we, who have been instrumental in your present precedence, should feel complacent pride in this formal closing of your professional school days, and appropriate that we give you, in a word, the after-life lesson.

This is a meeting of mingled emotions. The feelings of pleasure and pain, rejoicings and regrets, come freely floating over us as we blend in our view the past and the present.

A longer or shorter span of years ago found you all with your minds made up for the study of medicine. Choices of a profession were decided by a diversity of motives. One of you thought he had a mind adapted, by its organization and culture, as well as by its tastes, for benefitting the world in this way—and, in return, receiving a competency, and so fulfilling his mission. Another thought of the fame it would bring, another the gain, another dreaded manual labor, and one had a great benevolent heart, whose every murmur was good to his fellow man, and took this way to do his duty. From these or some other, *all* of you found yourselves deciding on a preceptor and completing your arrangements for beginning your studies. Does it come back to *some* of you now how difficult it was to complete these arrangements—of the obstacles rising mountain high, soul-depressing and unapproachable?

To one there stood in the way a penurious father. You had no father; and you, an affectionate and dutiful son, had a dear old mother, and good, kind, dependent sisters, and none to help. Others more fortunate had none of these.

Then came the first discouraging days, when sphenoidal and ethmoidal terrors haunted the sleeping hours, and fleshless fingers met languid responses to their bony beckonings, and sulphur seemed created for a new terrestrial use. Often, when hours had been consumed in perfecting anatomical details, and it seemed as if every fact and idea were in place and ready for use, a question from your preceptor was sufficient to dissipate the whole; and after strange and fruitless search after the lost ideas on the bare walls, you were compelled to *review*. This was one of the times when you came near "giving up and coming down." Perhaps the next was more successful, and these dark days gave way and the genial and happy days of student life, by degrees, took their places.

Then, when the last thread was worn and the last dollar mateless, by a bold stroke of pecuniary strategy, you found yourself prepared to resume your studies—and so, by these interrupted, determined and unyielding efforts, you at last found yourself selecting a school for your attendance. Here have you come, and by incessant toil, hazzard of health and even life itself, you find yourselves pronounced worthy to assume the duties and receive a recompense for the toilsome and perilous labors so freely and liberally given to your chosen profession. That I do not exaggerate the risks attendant is too deeply impressed upon your minds in one of your number's having paid the penalty for his assiduity with his young life.* He had arrived at an age when the cares and anxiety of fond parents seemed about to cease in the glowing hopes of budding manhood; but escape was not given him from the dangers of his own devotion, and he now rests silent forever. Peace to the dear boy.

It is now and here at the end and consummation of your long deferred hopes, that we feel called as a Faculty to confer, and you as our honored pupils to receive, tokens of our confidence and fraternal regard. Truly it is a time for you to feel happy and receive the commendations and congratulations of your friends.

Your forty months of pilgrimage brings you to the mount where you see the tedious, turbulent trials of the past from which you have escaped, and view the promised land; and as you look back along the time through which you have buffeted the hard way, your heart flutters for *very anxiety*, lest the past undo itself. You feel cheered as you contrast this with the future. Dry, dusty subserviency to books and masters is to be exchanged for the duties, dignities and confidences of the new position. It does look beautiful and inspiring. It gives the world a *new* glow, *home* seems better, and happier to receive you as a physician, friends more cordial when welcoming you as Dr., and the many preferences in life are sure to follow. The promised land spreads beautifully its broad and velvety expanses, laid in liquid silver reflecting the azure, deceptively seeming the true way to the etherial, dazzling and delighting your impatient spirit.

You are about to assume the full responsibilities of actual life. Hitherto you have acted a subordinate part. In starting in this great experiment, you cannot tell before hand *what* will happen to you. It may be *good*, it may be *evil*. I take the fact that you stand here this evening at the end of a long,

* Chas. S. West.

stern and perplexing course of study, most of you the honored instruments of your own success, as speaking much of hope for you. It gives me the *largest* confidence to see the first efforts of a man's life hedged about with thick set difficulties, and while he *occasionally gives up*, *never comes down*, until at last he breaks through them all, and every day gets richer in the world's experience and emoluments.

The great North-west is a fruitful field for observation, in this contest with circumstances. Here, as in other newly made countries, the boy must make his *own* battle with the world, and especially those pursuing the learned profession, or, in the father's language, playing gentleman. This is rendered easy in his eyes by the facility in getting funds in a very honorable way, viz: school teaching. How many of you have not taught? I venture three out of every four have. We have not arrived in our western civilization to that generally diffused competence to be found in older, more staid, and less enterprising communities. Here, if one of our yeomanry makes a success and wealth pours into his lap, he sits down and calculates the acres it will buy, and not the sons or daughters it will educate. What, spend a thousand dollars in collegizing one of my boys? That would furnish a feather bed, cow and freedom suit to the other fourteen.

This will undoubtedly change, as the revolving years smooth the hillocks and dry the morasses of our prairies, and replaces them with waving beauty and substantial prosperity. Our students will come to us more fully supplied with the comforts and luxuries of life, but it may be *not* with the necessaries. Money in profusion is not one of the necessaries. It is one of the DESTROYING ANGELS, and seldom leaves its door-posts bloodless. Rather let me say *poverty* is one of the essentials. Poverty is an element of strength, wealth of weakness. Poverty is a tonic, wealth an anodyne. One supports, the other paralyzes. I do not think I exaggerate, when I say the success of those born in affluence is the exception. There is something so enervating in having everything served to you. Every want is supplied at the word; the breath of asking is the price of anything All mental effort or enterprise being ignored and abandoned, the individual degenerates mentally and morally, as certain as must the unused limb. Exercise strengthens, and we understand the philosophy of it; and we know that he who is cradled, reared and disciplined by poverty, will present the bold, honest, manly independent original. The necessities of his situation develop these qualities. Make a young man poor, and the very effort of his inventive genious in striving to force himself against the current, will give more exercise, and more fully develop his mental power than anything beside. The goal is placed in the stream, the rich above and the poor below it. The favored float to it, and the lowly labor to it. The toiler has this advantage, though, that he is in *little* danger of *passing* it.

Don't understand me to mean, that all you have to do in life to *succeed*, is to be *poor*. If any of you are in the possession of any considerable of the world's wealth, you needn't go home and dispense it, with the idea of taking a fresh start in life with superior prospects of success. It is possible for one to be poor every way, to be a very indifferent fellow surrounded by all the blisters poverty brings.

The point aimed at in what I have said is, that other things being equal, the boy raised where honest necessity awoke him in the morning, and a frugal enterprise busied his hands during the day, and who slept the refreshing sleep of industry at night, bids fairer to become one of the world's cherished, than he whom pampering pride and luxurious prodigality too often fit for the fate they receive — *merited forgetfulness.*

There are students in our classes, who are worth their thousands, and yet my noble boy who paid your last greenback for the parchment you now hold, had I the power I wouldn't let you change places with *any of them.* Poverty is often frightfully unhandy and disagreeably inconvenient, but not where you are now. I have harge sympathies, but I

don't pity you one particle. Had I the power, I wouldn't give you a penny. I would let you *make* the money to pay your first board bill, with the first proud triumph of your art. Several weeks' unpaid bills are great energizers. You are not likely to complain of having your rest broken, or speak anything but courteously to patients when calling for your services, if you need theirs.

Jean Paul says, in speaking of the penury of his father's early life: "I cannot but choose to say to Poverty, 'Be wel come, so thou come not too late in life.' Riches weigh more heavily upon talent than poverty. Under gold mountains and thrones lie buried many spiritual giants. When to the flame that the natural heat of youth kindles the oil of riches is added, little more than the ashes of the Phœnix remains, and only a Gœthe has had the forbearance not to singe his wings at the sun of fortune."

Soon the ties that have bound us so kindly and agreeably together, *will* be *severed*, and you find yourselves engaging in the real profession. It is an interesting and pleasing portion of your life. The culmination of your past hopes seem now about to be realized, and you to assume the role for which your studies fit you. Hope and distrust now begin to mingle. There are few in whom the *former* does not *predominate*, and yet when we consider the fearful burden implied in the very name Physician, there are few who do not feel the latter. I should feel but little confidence in a person who was all self-assurance, and stepped boldly and unabashed untrodden ground. There will be much that will be new, and much that will gratify. Your first successes will be models. The first dossil of lint that you carefully place in an aching cavity, followed by immediate relief, you will feel like shouldering your whiting bush and making all the fences S. T. 1860, X. oil of cloves and morphine.

You will be employed distrustingly by your first patrons, *perhaps* from *necessity*, and *then* will be your *time* for extra efforts. If these efforts *are* crowned with success, your triumph will be the more perfect, and you will find a more secure place in their confidence and affections. Nothing is half so grateful as to be agreeably disappointed, and they will often make you amends for their distrust, in the most *lavish* expenditures of future friendship.

This leads me to say, never be discouraged by trifles. Now, were I to define trifles, I might surprise you in the extent to which this definition applies in this connexion. It means the lack of practice for a year or two in any grateful or flattering amount. None of you must imagine that there is any place with a practice ready for you, all collected to your hands, enough and to spare, and that being overrun with practice is to be one of your first burdens. The contrary is *much* more likely to be the case, the lack of it. It is to be confessed, however, that the experience of the graduates of our institution, your alma mater, furnish much grounds for flattering hopes. I say it here, and deliberately, that there is in my opinion no institution in our entire country, the graduates of which are so uniformly successful. They almost invariably succeed, and that from the moment of their locating. You may account for it, as you think; I account for it that they are the honorable sons of industry, self-made, self-reliant and noble. Much as this success flatters the vanity and sustains the pride and purse of its possessor, I cannot think it best calculated to develop your resources. Uniform success is almost as powerful a narcotic as wealth. It is apt to render you indolent and indifferent. In addition the usual leisure of the young practitioner gives time for the careful study of the acquired cases. You are much *less* liable to fall into routinism where you form your habits of study and practice in this gradual manner. It is often a misfortune to be too successful in the beginning, though it is not appreciated at the time, and never objected to.

Another of the things that should not discourage you, is that of not being invariably successful in your treatment of cases. Don't infer from this, that the loss of a life or failure to cure disease, are trifles in themselves, but they assume that character, when they, as natural consequences to be expected in an imperfect science, are *allowed* to deter you from your legitimate calling. The first patient you lose will come as a death knell to your heart, as well as ears, and should others speedily follow, the effect will be *startling* to your professional nerves, and the questions, havn't I missed my calling; isn't this an indication of my unfitness; haven't I mistaken the pill bags for the peg hammer, scalpel for the shears, the catline for the plough handles? will be disturbing thoughts in your quiet hours. The question is the answer. If you never *ask* the question. I will answer it now. I say yes if, you do ask it, I say no. Your sensitive conscientiousness is the best proof that the lives, healths and interests of your patients are well in your hands. It shows most clearly that you have a big professional heart, throbbing with the feelings of honor and humanity. Don't lay aside your mantle for causes of this kind. Many times your sympathies and anxieties will be brought up to the point of distraction, and the death of your patient will be little less than death to you. One, perhaps, of your earliest and firmest friends, between whom and yourself have grown interests the most sacred, ties the most tender, whose words are all kindness, whose deeds are all blessings, and as years pass the confidence grows until you next to God hold the supreme faith and when the grim monster grapples rudely and visibly at the tender heart strings you shudder and sicken, and I don't blame you. But *don't falter*. Do your duty faithfully, and abide the result.

Another discouraging and trying one of these harmless annoyances, is competition with quacks. There are few in the list more discouraging, than to see yourselves after having given the finishing touch to your education, commended by your teachers as worthy a place in the public esteem, located beside a full fledged scion of this discriminating craft. After you have labored faithfully and successfully for a time, perhaps, and the results of your practice begin to show gratifying evidences in friends and funds, suddenly you find yourself confronted with one of the aromatic gentry. It matters little what he professes. He will not fail to have followers, and you will be surprised and disgusted at the avidity with which the crumbs of comfort *he* deigns to drop for some hopeless consumption or corroding cancer, are snatched up. This will not be among the ignorant and uninformed. It will happen in the very midst of wealth and respectability. You hear nothing but the praises of the *newly* arrived. The air is filled with the buzzing and roaring of his friends, who flock around him as flies around new honey, and usually with the same result—getting badly stuck. You are apt to imagine that your prospects are dimming; that you are not appreciated; that if men of that cast succeed, what need of qualified men? that the world is made up largely of fools, and *various* other *doubtful* and inexpedient conclusions. One thing you may conclude, and that is, that there is no department of man's concerns about which he is so superstitious as the health of his body. He can decide where his interests lie on *any other* questions, with surprising accuracy, but the moment you begin to touch his weak point—his health, he is at the mercy of the wind. A man's health is his blind side; any one can come up to him there.

Quacks have come thousands of miles to this city, to my personal knowledge, to *lay their hands* on the *sick*, that they might be healed. And the subjects of these modern miracles didn't live in the miry precincts of "Kilgubbin." They do business where property costs five hundred dollars a foot, and reside in the most fashionable part of the city. This modern Savior went home with his pockets well lined, and his patient went in search of another.

They don't need that second sermon, preached by the minister who took for his morning text: "Ye are the children of the devil," and by a ludicrous coincidence, took for his afternoon: "Children, obey your parents."

This will seem *very*, *very* hard to you, and it is natural it should. But you must know that there will be quacks as long as there are ignorant and superstitious people to employ them; and from the constitution of the human mind, we fear in butterfly, or grub, they will last till the end. The most you will have to do, is to be slow to anger, and restrain the natural impulse you would have to garrote the hypocritical cosmopolites every time you meet them. These gorillas, when they have devoured what they can get with you, will seek prey some where else; some of your humiliated friends will come back to you well cured, and some will wait for the next.

These embrace but a small part of the embarrassing discouragements you will find planted in your path. You will have to run the gauntlet of gossip, and those two-edged swords placed in some mouths will pierce you to the very marrow. It will not depend on your own conduct, whether you escape this. The public servant who is *not* slandered, is below contempt.

You must not imagine that from these troubles which you will encounter, that there are not many bright, sunny spots on your future journey; that your *pleasures* are *all* things of the past. There is much, *very* much in the daily life of a devoted, earnest, true physician, full of sanctified enjoyment. You must not feel, if you do not prosper at first, that you will not succeed *at all;* that if you lose some cases, that you will not *save* some; or that because you meet some impostors, there are *nothing else.*

There is one thing—if you have a *human* organization—that will be most gratifying to you, and that is the *confidence* of your *patients.* When you have once thoroughly gained the confidence of your patrons, and see as plainly as they show, the strong and abiding faith they have in your skill as a physician, as well as honor as a man—when no word, deed or intimation from them, *ever* seconds an appeal to any one better--when you come to be regarded as the friend as well as the physician of your families, and are taken into their trust as truly and perfectly as you ought to be—when you reckon your friends in the household by the number of it—when the oldest speak of you by day, and the sick children cry for you at night, to be made the subject of pleasant allusion at your next visit—when the little ones gather about your knees—when any little comfort contributed to you is the pride of all—when you merit and receive this confidence, it will be the anodyne for the poisoned minds of neighborhood anacondas, the soft place to rest the wearied limbs, the nectar to your parched spirit, the star of hope and comfort when the night of despondency gathers gloomily about you.

And then, if I mistake not the cast of mind and character of those who are now before me, whose diligent devotion to the principles we have endeavored to elucidate, has been the cheeriest thought that crowned our labors, if I mistake not, you cannot fail to feel a longing desire to try and test for yourselves the truth we have taught, and watch the development of facts in connexion with our art, and investigate and apply new ones. Here will be one of your most constant sources of pleasure. It will be your daily duty, your hourly occupation. It is *in* this, and *for* this, that you must leave father and mother, wife and children, and follow where your profession calls. It is this which will largely test your fitness for your chosen calling. If you should find a constant antagonism between yourself and the practice of medicine, a feeling of distrust and uncertainty, and enduring apathy, I would not tell you *this* was one of the *trifles*. There would be written on your heart's inner door, "*notice to quit.*" The sooner you can dispose of your stock in trade, and sweep from your memory with the scrub brush of conscience the last memories of medicine, the better for the human family. You have embraced a calling that requires, and successfully to fellow, *demands* all the judgment, genius, perception and engrossed vigilance of your entire mind; and to give a halting, unwilling, distracted, pecuniary, hair-brained consent in following this our noble calling, is sacrilege in its worst sense, and deserves to have the tables of the money changers overturned and they be cast out of the temple.

What can be worse than being wedded to a profession which is constantly burning your soul. You are married, not mated, when the desire for divorce constantly haunts you, making your life miserable, except when dwelling on the sordid ends to be gained by it. Knock off the shackles and don't allow yourself to be dragged down by any false pride of position. There is a place for you; by all means find it; rectify your mistake, and leave the profession better for your absence.

These are some of the things which will unbend your bowed frame, and cheer with the happy joy of summer sunlight the way of your labor and love. Much of the pleasure derived in this life depends on the individual rather than extraneous circumstances, none the less of you. It often depends on the cornea we look through, whether the horizon look dark, blue, or bright. It takes but little coloring there, to mantle the world, and don't go to renovating the world while the fault lies on the proximal side of your visual organs.

Allow me in the few remaining moments of our interview, to give you two or three practical suggestions as to how you can avoid the failures and disappointments, and get the good.

In the "Pursuits of Literature" the advice is given to young men, "dare be ignorant of many things." I offer the same to you with this addition, "BUT NOT OF YOUR PROFESSION." Let nothing short of a perfect knowledge of our science satisfy you. Here is the master key to success. Herein you have your fortune in your own hands. In this you can diminish the distance between you and those whom the greater advantages of education or brilliancy of intellect the world would set before you. HOW CAN I do this? you ask. I have friends to make, business to attend to, the cares of a family—am distracted with anxieties for my patients, the wherewithal to keep life's wheels moving must be raised, and it seems impossible that I can give the needed time for the extensive reading necessary for such erudition. Every one of these reasons you enumerate why you cannot read, is one of the most urgent I could offer for the suggested course. How can you make friends better than by gaining worthily the reputation of a thoroughly educated physician. How so well prepared for that business as with a mind plethoric with the experience and facts of the Sages? How care for a family so well as on the *funded* effect of knowing your business? Time is not wanting. You can have all the time you need, by subtracting it from your idle moments, and never miss it. Reading is relaxation from practice. It is the best and most interesting recreation for you in the world. Any of you can save one hour a day, and many of you at first, six, and not diminish the time for the duties or pleasures. You waste time enough if rightly employed to make you the greatest wisacres in the land. If the moments *needlessly* wasted in the life of every professional man were gathered together, some would be surprised to find one-fourth of their time idled away. If the books he *could* read and *does not*, were piled up at the end of the year for him, he would consider book cases a *tax*. No excuse on the want of ability to purchase the needed volumes. You can get them just as easily as you can get time to read them, and in precisely the same way. "In all your gettings get wisdom." It is this that will distinguish you, and raise you above the common level. It is this more than any quality of affability or brilliancy, that will secure you the respect of all good and intelligent people.

Cultivate thorough honesty and frankness in all your intercourse with patients and professional friends. What so becoming, so desirable in one who takes such a place in the affections and interests of those committed to him, as thorough *integrity* and *candor*. To have your patients feel that you are the unselfish friend and honest counsellor, the candid communicant of all, whether good or ill for them, will give them the kind of trust in you which will give your words the weight they merit. How much there is to admire and desire in one whose thorough integrity and candor are proverbial qualities. The want of them *may* give you temporary reputation, or *delay* the necessary pang surely impending; but be assured it will not fail to injure you when the results of your match with death can no longer be concealed. *Tell them* the *truth* every time they ask for it. Let them know that their concerns are as sacred with you as with themselves, and make up your mind deliberately to die rather than betray them. You are very peculiarly related to your patients, and it becomes a part of your profession to guard their confidential communications as sacredly as you would the interests of your own soul. These often come when your manhood and every impulse of a generous nature enjoins upon you the strictest confidence; but there is one weightier than all, and that is your professional honor. Many a household has been rent asunder by the unwarranted indiscretions of a garrulous physician.

Be kind and cheerful with your patients—kind without offensively patronizing them, and cheerful without being light. How much it soothes the sharp pangs of suffering to have kind and gentle words from the sympathising physician. It makes his hand softer, to smooth and correct the shattered fragments. Every twinge seems lighter for these cheap sedatives. Your sympathy needn't unnerve your skill. You needn't gush into tears at every sight of suffering. That would be worse than none. Your first duty is to bestow your skill, and the next to give words of comfort. First stay, and then soothe. Kind and considerate sympathy is entirely compatible with the *highest* skill, and the coolest and most determined resolution. You can staunch the crimson flow with *one* hand, and have one left to chafe the aching brow. Don't let this sympathy be exclusive. Remember those whom you always have with you, as well as those who add to your income. Let it not be of the kind only shining when *gilded.* But *rather*, be a friend to those who need and cherish your kindness.

Then I might speak of and tell you how necessary industry was to your good fortune; that "labor, the symbol of man's punishment, is the secret of his happiness;" of equanimity of temper under his vexatious troubles necessarily connected with your chosen life; of the incessant care you should exercise over your patients; and, in fact, occupy your hours instead of minutes, in their detail.

One thing more—don't change. Get your minds well fixed on a location for good and substantial reasons, and don't leave it. A floating, shiftless, undeciding, dissatisfied physician, is the most dilapidated, disagreeable, unprofitable thing I can think of. They act as though they were determined to have it all, and so circulate their valuable services. Birdofreedom Sawin, Esq., has it:

> "Change just for the sake of change,
> Is like these big hotels,
> Where they're always changing plates,
> And let ye live on smells."

I am admonished that as the weeks have passed rapidly away since our first meeting, so the few moments allowed me give but little time for telling you everything which experience intimates would be useful to you. It is all included in *Education, Honesty and Sympathy.*

The past has been to us a winter of unusual interest and pleasure, and the agreeable associations thus formed make the present parting the more painful. This, the twentieth annual session, has been the *most* prosperous our institution has ever known, more students having enjoyed its advantages than in any previous year; and it has been the constant remark of your teachers, that the attention and assiduity of the class, as well as their intelligence and scholarly attainments, conferred lasting honor upon it as a class and us as a Faculty. But this compliment, I would say, we cannot promise you longer than twelve months.

We feel, in sending you to the world as the representatives of your alma mater, we commit much to you. Not only are our interests, but those of humanity, are in your hands. We say you are prepared for high and noble positions, and have solemnly pledged our faith for your qualifications. Shall we, shall the world be disappointed? Shall our cheeks ever feel the blush of shame for disgraceful deed by you, our beloved pupils. All the attentive months you have so patiently and willingly sat as learners before us, all the midnight hours you have scrupulously devoted to your intricate studies, all your manly faces answer NO. You go out the largest delegation we have ever donated to the world, and you go at an interesting time of its history. Your services are needed on many a bloody field, where a brother calls you to staunch fraternal blood which his own hands have shed. Our patriotic and surgically-abused soldiery call on you to save them from those who *should be their friends.* Louder comes the call from the thousands whose freezing blood fastens their helpless bodies to the crispy earth, to save lest they perish.

To such as feel the calls of our blessed country paramount, who will take their lives in their hands and peril all for her, we say, go, and when the number so gone renders our services as teachers needless here, we'll close the doors of your alma mater, hang out the stars and stripes and follow you, while duty calls, or life remains.

Gentlemen, we part. For the very many acts of courtesy we have received at your hands, we would express to you our deepest thanks, and may the happy hours we have spent together, often come to us in after day thoughts, as fragrant incense burning upon the altar of friendship, ascending to the very heaven of heavens.

May your lives be the perfection of professional success, and draw from some truthful biographer, the glorious and undying commendation so worthily bestowed by the prince of historians, on the prince of earthly princes: "As long as he lived he was the guiding star of a great and noble profession, and when he died the little children cried in the streets."

Gentlemen, go; God bless you. Good bye.

